# Parental reporting of adverse drug reactions in South Africa: An online survey

**DOI:** 10.4102/phcfm.v13i1.2880

**Published:** 2021-09-29

**Authors:** Shavani Pillay, Mwila Mulubwa, Michelle Viljoen

**Affiliations:** 1 School of Pharmacy, Faculty of Natural Sciences, University of the Western Cape, Bellville, South Africa

**Keywords:** adverse drug reactions, spontaneous reporting, patient reporting systems, parental reporting, pharmacovigilance

## Abstract

**Background:**

The high incidence of adverse drug reactions (ADRs) in children is of global concern. Enhancing the reporting of ADRs could contribute to making safer medicines available to children.

**Aim:**

To assess parents’ awareness of reporting ADRs and their knowledge on the reporting procedures in South Africa.

**Setting:**

South African parents with online access.

**Method:**

A quantitative descriptive study was conducted based on an anonymous voluntarily web-based self-administered questionnaire that was distributed through Facebook® and LinkedIn™ to parents in South Africa.

**Results:**

The questionnaire was completed voluntarily by 206 respondents. The majority of participants (70.9%) were aware of the term ADR. Significant associations between not being aware of the term ADR and single marital status, lower education level, not having private medical aid and accessing public clinics for medical services were found. The majority (66.5%) of participants did report an ADR to a healthcare professional whilst only 15% reported it to a product manufacturer. More than half of the participants (58.7%) knew how to report ADRs whilst 72.8% knew what type of ADRs to report. Almost a third (32.5%) did not know where more information on ADR reporting could be found or how ADRs could be reported (31.5%).

**Conclusion:**

The majority of the respondents were aware of the term ADR, indicative of a good knowledge basis on which ADRs to report and the importance of reporting ADRs. However, gaps in the respondents’ knowledge were identified which highlighted specific groups of individuals to be targeted to increase ADR awareness and improve the knowledge on the reporting process.

## Background

Medicines are developed with the intention of helping patients, but they may be harmful to the patient by causing adverse reactions.^1^ The monitoring of adverse drug reactions (ADRs) to ensure patient safety is a critical component of pharmacovigilance.^2^ Pharmacovigilance is defined by the World Health Organization (WHO) as ‘the science and activities relating to the detection, assessment, understanding and prevention of adverse effects or any other drug-related problem’.^3^

Studies conducted throughout the world found that ADRs constitute over 6% of all hospital admissions and are amongst the leading global causes of morbidity and mortality.^4^ The WHO defines an ADR as:

[*A*] response to a drug which is noxious, and unintended, and which occurs at doses normally used in man for prophylaxis, diagnosis or therapy of disease, or for the modification of physiological function.^5^

ADRs result in longer hospital stays and higher costs incurred for the patient and the healthcare system.^6^ Research shows that in some developed countries, up to 20% of their hospital budget is spent on managing ADRs.^7^

A major concern is the high incidence of ADRs in children. Numerous medicines have not been adequately tested and approved for use in children.^8^ This results in off-label use of medicines in children which is linked to an increased risk of ADRs.^9,10^ Pharmacovigilance is an essential component of ensuring the safe use of medicines in children.^8^ The foundation of pharmacovigilance programmes is the reporting of ADRs spontaneously by healthcare professionals.^6^ Spontaneous reporting allows for unknown or uncommon reactions to be identified and can contribute to making safer medicines available to patients by facilitating the withdrawal of potentially unsafe medicines from the market.^7^ Patient reporting systems and allowing for the general public to report directly to health authorities, also referred to as consumer reporting,^11^ provide the public with the opportunity to be more involved in their own care. These have existed in many countries, including the United States (US), Canada, Australia, the Netherlands, Italy, Sweden, the United Kingdom (UK) and more recently Norway.^2,6,11^ Patient reporting systems were introduced in European Union (EU) legislation to improve medicine safety and has been seen as a valuable contribution to protecting public health.^12^

Patient reporting is not actively promoted in many countries because of financial constraints and a lack of resources.^13^ In South Africa, a spontaneous reporting system is used, in which healthcare providers are responsible for reporting suspected ADRs to the National Adverse Drug Event Monitoring Centre (NADEMC), a unit of the South African Health Products Regulatory Authority (SAHPRA).^14,15^ The current system for patients to report suspected ADRs directly to the NADEMC or SAHPRA are poorly designed. The majority of the patients are unaware about how these processes work, but they can report to pharmaceutical manufacturers, either telephonically or via the respective pharmaceutical company website.^14^

Under-reporting of ADRs has been recognised to be a common shortcoming of pharmacovigilance programmes in South Africa as well as internationally.^6,7^ Hazell and Shakir (2006) reviewed spontaneous reporting systems from 12 countries and found that on average 94% of ADRs were not reported.^16^ Under-reporting prolongs the detection of ADRs and may result in increased death and suffering in patients.^4^ As medicines are intended to benefit patients, obtaining information directly from patients plays a key role in identifying new ADRs.^17^ Research has shown that patients worldwide have substantial interest in the safety aspects of medicines and allowing them to report ADRs has offered a unique approach to pharmacovigilance.^1^

Because parents have a typical caring and protective role, they could play an important part in detecting and reporting ADRs in children. Evidence suggests that parental reporting provides several benefits for pharmacovigilance, including increasing the rate of reporting of ADRs and identifying previously unknown ADRs in children.^18^ In countries with patient reporting systems, parents were found to be unaware of their role in reporting ADRs.^19^ This could mean that the awareness of parental reporting in countries without a patient reporting system may be lower. The contribution of parental reporting to pharmacovigilance in South Africa can be substantial. However, parents’ awareness and knowledge of ADR reporting and the process involved in it have to be considered first.

The aim of the study was to evaluate and assess the awareness and knowledge of parental reporting of suspected ADRs in South Africa.

## Methods

### Study design

A quantitative descriptive study was used to conduct a survey, which was based on an anonymous online self-administered questionnaire, amongst voluntary participants to assess their awareness and knowledge of reporting ADRs.

### Study population

The survey was conducted on parents over the age of 18 years and living in South Africa. Male and female parents as well as parents of adopted children and/or step children were included. Parents who could not read or understand English, those who were minors (under the age of 18 years), and those who were South Africans, but lived abroad, were excluded. Parents of children older than 18 years were also excluded.

### Sampling

This was an all-inclusive convenience sample of parents who responded voluntarily to the online questionnaire.

### Data collection

A web-based self-administered questionnaire was constructed using Google Forms and these were distributed online to parents in South Africa. The link to the questionnaire was distributed on social media platforms, such as Facebook® and LinkedIn™ between July 2018 and August 2018. The questionnaire comprised three sections that covered demographic information, ADR awareness and knowledge, and views on ADR reporting. It consisted of 28 closed-ended questions and four open-ended questions. Thematic analysis was performed on the open-ended questions to identify themes within the data.

### Pre-test and validation of instrument

The researchers, together with colleagues and subject matter experts were involved in the questionnaire design and development to ensure face and content validity. The questionnaire was based on reviewed literature and questionnaires on the same subject matter.^20,21,22,23^ The questionnaire was piloted before implementation by administering it to five volunteers who were similar to the target population but were not included in the main study.^24^ Necessary changes were made thereafter to improve the structure and clarity of the questionnaire.

### Data analysis

The data collected was coded and entered into a Microsoft® Office Excel spreadsheet. Data was analysed using Statistical Package for the Social Sciences (SPSS) (IBM® SPSS® Statistical software, version 23). Descriptive statistics were used, and the data was summarised using percentages, frequency tables and bar charts. Associations between categorical variables were determined using the Pearson Chi-square (χ^2^) test and relationships were considered statistically significant if the *p-*value (α) was ≤ 0.05. Adjusted *p*-values (Bonferroni correction) were calculated for multiple comparisons.^25^ For each of the multiple comparisons such as in the case of employment area, the critical *p* –value (α) in this study (0.05) was then divided by the number of comparisons being made to set a new stricter significant threshold level as a post-hoc test for probability to control possible false positives and negatives.^25^

### Ethical considerations

Ethical approval was obtained from the Biomedical Research Ethics Committee of the University of the Western Cape (Reference Number: BM/18/4/5) prior to the online survey distribution. Participants were invited and informed on the social media platforms what the survey was about. If they agreed to take part in the survey, they were requested to click on a specific link which opened the first page of the questionnaire pertaining to the informed consent. If they disagreed with the following statement, the survey was terminated: ‘The study has been explained to me in a language that I understand, and I freely and voluntarily agree to participate’. No email addresses or personal identifiers were requested or captured.

## Results

### Socio-demographics

A total of 206 parents completed and submitted the online questionnaire voluntarily during July 2018 – August 2018. The detailed socio-demographics of the respondents are summarised in Table 1. Overall, 75.2% (*n* = 155) of the respondents were female. There was representation from each of the nine provinces, although the majority (68.4%, *n* = 141) of the respondents were from Gauteng. A large percentage (48.5%, *n* = 100) of the respondents were in the age category 31–40 years. A greater number of respondents were married (73.8%, *n* = 152) and 45.6% (*n* = 94) reported to having two children.

**TABLE 1:** Socio-demographic characteristics of participants.

Characteristic	Number of participants	%
**Gender†**		
Male	50	24.3
Female	155	75.2
**Age (years)**		
18–30	33	16.0
31–40	100	48.5
41–50	60	29.1
> 50	13	6.3
**Province**		
Eastern Cape	12	5.8
Free State	6	2.9
Gauteng	141	68.4
Kwazulu-Natal	14	6.8
Limpopo	5	2.4
Mpumalanga	4	1.9
North West	4	1.9
Northern Cape	3	1.5
Western Cape	17	8.3
**Marital status**		
Single	26	12.6
Married	152	73.8
Divorced	20	9.7
Separated	6	2.9
Widowed	2	1.0
**Number of children**		
1	59	28.6
2	94	45.6
3	35	17.0
4 or more	18	8.7
**Highest level of education†**		
Did not finish school	6	2.9
Matric certificate	41	19.9
Diploma	68	33.0
Degree	75	36.4
Other	15	7.3
**Primary area of employment** ‡		
Student	3	1.5
Unemployed	11	5.3
Automotive industry	15	7.3
Education and training	35	17.0
Financial services	35	17.0
Healthcare	40	19.4
Information technology	10	4.9
Legal services	8	3.9
Wholesale and retail trade	9	4.4
Other	32	15.5
**Medical aid**		
Yes	177	85.9
No	29	14.1
**General medical services†**		
Private doctor	177	85.9
Private nurse	1	0.5
Pharmacy	15	7.3
Public clinic	12	5.8

† , did not fill in on the survey: *n* = 1;

‡ , did not fill in on the survey: *n* = 8.

Most of the participants indicated that they had a qualification post finishing school (76.6%, *n* = 158), with the highest level of education reported as a master’s degree. The primary area of employment was diverse with healthcare (19.4%, *n* = 40) being reported the most, followed by education and training (17%, *n* = 35) and financial services (17%, *n* = 35). The majority of respondents had private medical aid (85.9%, *n* = 177) and made use of private physicians (85.9%, *n* = 177) for their medical services and needs.

### Adverse drug reaction awareness and knowledge

It was established that 70.9% (*n* = 146) of the respondents were aware of the term ADR before completing the questionnaire, whilst 29.1% (*n* = 60) were not aware of it. Although many of the respondents were aware of the term ADR prior to taking part in this investigation, it was important to see if certain socio-demographic factors could have played a role in this awareness more than others. This could potentially identify possible areas where more focussed health education, training and awareness can be encouraged in future public health endeavours. Significant associations existed between most socio-demographic variables and awareness of the term ADR. A summary of associations is shown in Table 2. Respondents with a post-school education (diploma or degree), private medical aid and access to private medical services were significantly more likely to be aware of the term ADRs.

**TABLE 2:** Associations between socio-demographic variables and awareness of the term ‘adverse drug reaction’.

Socio-demographic variables	Aware – Yes	%	Not aware – No	%	Pearson Chi-Square (χ^2^)	*p*
**Marital status**						
Married	113	74.3	39	25.7	3.4	0.066
Unmarried (single; divorced; separated; widowed)	33	61.1	21	38.9		
**Education level**						
Post-school education (diploma; degree; other)	128	81.0	30.0	19.0	32.3	< 0.001
No post-school education (did not finish school; completed secondary school Grade 12)	18	38.3	29.0	61.7		
Employment area*						
Student	1	33.3	2	66.7	2.1	0.144
Unemployed	5	54.5	5	45.5	1.6	0.209
Automotive Industry	7	46.7	8	53.3	4.8	0.030
Education and training	29	82.9	6	17.1	2.8	0.094
Financial services	27	77.1	8	22.9	0.7	0.393
Healthcare	36	90.0	4	10.0	8.6	0.003
Information technology	7	70.0	3	30.0	0.01	0.931
Legal services	6	75.0	2	25.0	0.1	0.809
Wholesale and retail trade	3	33.3	6	66.7	6.6	0.010
Other	19	59.4	13	40.6	2.6	0.106
**Medical aid**						
Medical aid	132	74.6	45	25.4	8.3	0.004
No medical aid	14	48.3	15	51.7		
**Access to general medical services**						
Private (private doctor; private nurse; pharmacy)	142	73.6	51	26.4	12.9	0.001
Public (public clinic)	3	25.0	9	75.0		

* , Adjusted *p*-values (Bonferroni correction) were calculated for multiple comparisons. Setting new threshold significance for each of the multiple comparisons, the critical *p*-value (α) was 0.05/number of comparisons. Only *p*-values < 0.003 were significant.

None of the employment areas, including being a student or unemployed indicated any significant association with being aware of the term ADR.

More specific details about the respondents’ knowledge of ADRs are reflected in Table 3. Many respondents recognised that all medicines can cause ADRs (*n* = 130) and overwhelmingly 91.7% (*n* = 189) indicated that access to information on ADRs contributes to improving patient safety.

**TABLE 3:** Participants’ knowledge of adverse drug reactions.

Question	Responses	*n*	%
What type of medication can cause ADRs?†	New medicines	35	17.0
	OTC medicines	47	22.8
	Complementary medicines (traditional, herbal, etc.)	16	7.8
	All medicines	130	63.1
Does the collection of information on ADRs contribute to improving patient safety?	Yes	189	91.7
	No	14	6.8
	No response to question	3	1.5

ADR, adverse drug reactions; OTC, over-the-counter.

† , More than one option could have been indicated.

**FIGURE 1:**
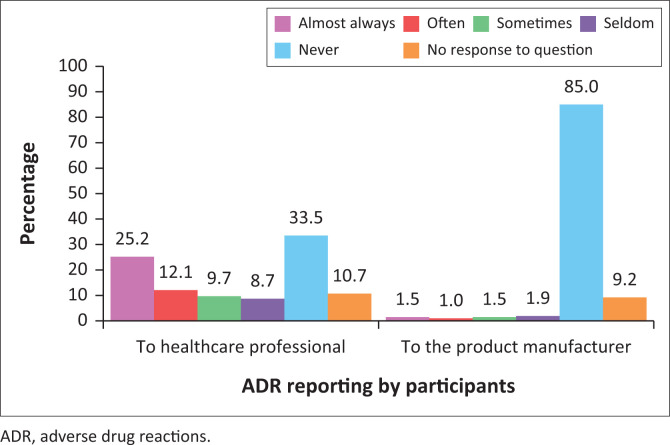
Reporting of adverse drug reactions to healthcare professionals and product manufacturers.

Figure 1 presents the reporting of ADRs by participants to healthcare professionals and product manufacturers (pharmaceutical applicants). A third of parents (33.5%, *n* = 69) never informed a healthcare professional about an ADR experienced by themselves or their child and majority of parents (85.0%, *n* = 175) never informed the product manufacturer about their ADR encounter.

Table 4 presents frequency counts (where more than one option could have been indicated) and percentage to questions regarding the ADR reporting process. When asked about where could more information on ADR reporting be found, a pharmacy was the selection for majority of the respondents (*n* = 114) with the remaining selections being a doctor’s surgery (*n* = 90), a pharmaceutical company (*n* = 83) and a hospital (*n* = 73). A small number of participants believed that only healthcare professionals should report ADRs (*n* = 32), similar to those that believed that only patients should report ADRs (*n* = 30).

**TABLE 4:** Participants’ responses regarding the reporting process or steps to be taken.

Question	Responses	*N*	%
Where can you find more information on ADR reporting?†	From a hospital	73	35.4
	From a pharmacy	114	55.3
	From a doctor’s surgery	90	43.7
	From a pharmaceutical company	83	40.3
	I do not know	67	32.5
To whom can ADRs be reported?†	Doctors	154	74.8
	Nurses	104	50.5
	Pharmacists	136	66.0
	Product manufacturers	183	88.8
	NADEMC	165	80.1
	No response to question	3	1.5
How can ADRs be reported?	Only by post	1	0.5
	Only by telephone	6	2.9
	Only by email/website	12	5.8
	By post, telephone, email/website	121	58.7
	I do not know	65	31.6
	No response to question	1	0.5
What type of ADRs should be reported?	Serious or life-threatening ADRs	30	14.6
	Uncommon ADRs	5	2.4
	ADRs not indicated on package insert	17	8.3
	All suspected ADRs	150	72.8
	Reporting is not necessary	4	1.9

ADR, adverse drug reactions; NADEMC: National Adverse Drug Event Monitoring Centre.

† , More than one option could have been indicated.

Regarding to whom ADRs can be reported, a large number (*n* = 183) of participants selected product manufacturers, 165 selected NADEMC, 154 selected doctors, 136 selected pharmacists, and 104 indicated that ADRs should be reported to nurses. It was surprising to note that more than half (58.7%, *n* = 121) of the respondents had knowledge of how to report ADRs (by post, telephone, email/website), whilst 9.2% (*n* = 19) were incorrect (only be post/ only by telephone/ only by email/website) and nearly a third (31.6%, *n* = 65) indicated that they did not know how to report ADRs. The majority (72.8%; *n* = 150) of respondents knew that all suspected ADRs should be reported.

### Views on adverse drug reaction reporting

The respondents’ views on public reporting are shown in Table 5. Almost all of the respondents (*n* = 204, 99.0%) believed that reporting of ADRs by the public is important, whilst only one participant (0.5%) believed that it is not important at all. The respondents were overwhelmingly (82.0%, *n* = 169) adamant about reporting ADRs in future and only three (1.5%) indicated that they would not consider reporting ADRs in future.

**TABLE 5:** Participants’ views on public reporting.

Question	Possible responses	Number of participants	%
How important do you think it is for the public to report ADRs?	Absolutely essential	152	73.8
	Very important	49	23.8
	Moderately important	3	1.5
	Not important at all	1	0.5
	No response to question	1	0.5
Would you consider reporting suspected ADRs in future?	Definitely	169	82.0
	Probably	23	11.2
	Possibly	10	4.9
	Probably not	2	1.0
	Definitely not	1	0.5
	No response to question	1	0.5

ADR, adverse drug reactions.

The respondents were requested to describe in their own words about which factors could motivate or prevent them from reporting ADRs experienced by their children or themselves. The most prominent positive theme that emerged was a social concern (*n* = 48). Participants were particularly concerned with helping others. Their responses were as follows: ‘I will not want someone else to have a bad experience’, ‘I would report it so that no other child or person goes through it’, ‘So my feedback can help other parents or people’. Other factors motivating the reporting of ADRs included severity of the reaction (*n* = 34), safety concerns (*n* = 16) and ADRs experienced by self/family (*n* = 14). Less frequent responses include product improvement, receiving feedback, receiving more information about the reporting process, fear/anxiety, increasing healthcare professional awareness, if the reaction is unexpected and if a causal relationship has been established.

Almost one third of the participants (32.5%, *n* = 67) indicated that nothing would prevent them from reporting ADRs experienced by themselves or their children. Some barriers reported by participants were process issues (17%, *n* = 35): ‘long hauled process of reporting’, time constraints (1.9%, *n* = 4): ‘just being busy and not having time to report it’ and no feedback or actions taken (4.4%, *n* = 9): ‘should no action be taken, I would feel less motivated to report it’. Some respondents indicated that they would be reluctant to report ADRs because the ADR was minor (4.3%, *n* = 11), or they were uncertain about whether the medicine caused the reaction (2.4%, *n* = 5). Less frequent barriers reported include forgetfulness, procrastination, fear of intimidation and condemnation, lack of awareness, lack of resources, lack of guidance from healthcare professionals and unapproachable medical staff.

Methods indicated by respondents to educate and inform the public about reporting include awareness campaigns through television (TV) and radio (*n* = 174), patient education by healthcare professionals (*n* = 171), information on product packaging/leaflet (*n* = 140) and published articles on ADR reporting (*n* = 110). Other suggestions included internet campaigns through social media, verified information via online parenting forums, and awareness campaigns at schools for parents to attend.

## Discussion

The concept of reporting ADRs in children is very important to consider. Despite the fact that the impact of ADRs on healthcare professionals’ workload and patients have become more prominent over the last two decades, the reporting of ADRs by healthcare professionals in South Africa remains low.^14^ This results in many patients, particularly children, potentially being exposed to medicinal products with an uncertain safety profile.^7^ Studies conducted through interviews in the UK, US, Australia, Canada and other countries, found that parents’ awareness of ADR reporting was low.^19,26^ This study contradictory revealed that majority (70.9%) of the parents were aware of ADRs and the importance of reporting them. In a much larger cross-sectional study in India where questionnaires were completed (*n* = 770) in hospitalised patients over four months, it was shown that 74.0% of the patients were aware of ADRs.^21^

In this study, as evidenced by the survey responses from these parents, they recognised that ADRs could harm people of all ages, that all types of medicines can cause ADRs and that reporting of ADRs can contribute to improving patient safety. Despite the infrequent reporting of ADRs by parents in this study, respondents had knowledge of where to find more information on ADR reporting and surprisingly, how ADRs can be reported. As reported in prospective paediatric pharmacovigilance study (semi-structured telephonic interviews) in the UK^10^ and in hospitalised patients in India,^21^ majority of participants displayed a positive attitude towards reporting ADRs and recognise the important role it plays within the healthcare system, which was also evident from the parents’ respondents in this study.

It is important to note that most of the participants in this study were well educated (76.6% completed post-school education higher than Grade 12) and employed, and the majority had medical aid (85.9%) and received general medical services from the private sector. This is in stark contrast to the results from a General Household Survey conducted from January 2017 to December 2017 which concluded that only 13.9% of South Africans had a post-school education higher than Grade 12 and that only 16.9% of South Africans were beneficiaries of medical aid cover.^27^

Studies conducted in India and Poland revealed that participants who lived in urban areas had more knowledge on ADR reporting compared to those that lived outside of the city.^20,23^ In a study conducted in Saudi Arabia, where a large percentage of participants (62.2%) were students or unemployed, it was found that patients were unaware of ADRs and ADR reporting.^22^ In this study, the percentage of students (1.5%) and unemployed (5.3%) were small and surprisingly no association was found with regard to awareness of the term ADR which could have had a different outcome if the sample population was larger.

Primary contributors of ADR reports are healthcare professionals although it is a concern for all.^28^ Research has shown that this responsibility should be shared between all parties and this was supported by most participants in this study, who stated that ADR reporting should neither be the healthcare professional’s nor the patient’s sole responsibility. As reported in previous studies,^10,21^ participants displayed a positive attitude towards reporting ADRs. In this study, the attitude of parental respondents was also positive. However, there was evidence of under-reporting of ADRs, with more than a third (33.5%) of respondents not reporting it to any healthcare professionals and unsurprisingly a larger percentage (85.0%) not reporting it to the pharmaceutical companies/product manufacturers.

Two crucial problems affecting ADR reporting were identified in this study. These include patients anticipating a complex process and having insufficient knowledge about the process. Previous studies conducted in the UK showed that after the aim and procedure were explained, parents were supportive of ADR reporting and found that the process was not complicated^26^. The findings from these studies suggest that in order to overcome under-reporting, patients’ knowledge regarding ADR reporting needs to be improved. If appropriate information is communicated to patients, they may report ADRs more frequently, thus contributing towards better management of medicine safety.^10,21,26^ This study overwhelmingly supports that the reporting of ADRs may be increased if sufficient knowledge is imparted to parents and access to relevant pharmacovigilance information is more readily available to contribute to improving patient safety.

In a worldwide survey based on telephone interviews, e-mail discussions and field visits, van Hunsel and coworkers^19^ concluded that information on ADR reporting needs to be disseminated using several methods and media in order to reach a larger audience. In Saudi Arabia, ADR reporting was promoted through educational campaigns and dissemination of flyers. Patients recommended that information can be provided through product labels and packaging as well as notices on regulatory authority websites.^22^ In this modern century, numerous information sources are available to the public to access and promote ADR reporting. These include health magazines, face-to-face wellness programmes, radio and television programmes, social media and various internet websites.^29^

It is important that all patients including parents be encouraged to report suspected ADRs and interventions should be made to improve the public’s knowledge regarding pharmacovigilance and ADR reporting procedures. This study identified opportunities for public health education and awareness to be implemented through various methods such as awareness campaigns through TV, radio, social media, at schools for parents, online parenting forums, education through healthcare professionals, product packaging on ADRs.

### Strengths of the study

Voluntary responses were received from 206 individuals with different socio-demographic characteristics. By using a web-based survey, a large number of individuals could be reached if willing to respond. Respondents could respond to the questionnaire at their chosen time and own pace. It was a convenient method to gather data with minimal costs. Anonymity was maintained through the online survey tool, which provided an opportunity for honest and unambiguous responses.

### Study limitations

This study had several limitations, particularly related to the study population. The questionnaire was only made available in English and therefore participants who could not read or understand English were excluded. The study methodology excluded the voice of the less literate and individuals in poorer communities who did not have access to internet and social media. Self-selection bias may have been introduced because of distribution of the survey on social media, which could have skewed the results of this study. Reliability coefficients for the questionnaire was not conducted. The majority of participants lived in Gauteng. Therefore, the results cannot be generalised to the larger population of parents in South Africa.

### Recommendations

Various pharmacovigilance awareness programmes should be conducted to encourage the reporting of ADRs by parents. Strategies to increase patient reporting should focus on frequent and feasible barriers to address. In addition to raising awareness, greater attention should be given to improving the public’s understanding of the reporting procedure, where and how to report and the importance of reporting ADRs.

More extensive research is required to evaluate the awareness, knowledge and views of ADR reporting by parents in all provinces in South Africa, including rural areas. Special efforts should be made to specifically target and educate populations identified as being less aware, to raise awareness of ADRs and the reporting process to individuals who have not finished school, have only completed secondary school, have no private medical aid or who visit public clinics for general medical services.

## Conclusion

This study suggests that these parental-respondents were aware and willing to report ADRs. However uncertainty as to who reports ADRs and to whom, difficulties with ADR reporting procedures, and time constraints were found to affect parents’ likelihood to report.

Respondents with a post-school education, having private medical aid and access to private medical services were significantly associated with being more aware of the term ADR contrary to respondents, having secondary or less schooling education, no private medical aid and attending public clinics for health services who were more likely to indicate that they were not aware of the term ADR before completing this survey.

The reporting of ADRs in South Africa may be increased if sufficient knowledge is imparted to parents and if access to relevant pharmacovigilance information is made readily available, thereby contributing to improved patient safety.
